# Safety and Efficacy of Degradable Starch Microspheres Transcatheter Arterial Chemoembolization as a Bridging Therapy in Patients with Early Stage Hepatocellular Carcinoma and Child-Pugh Stage B Eligible for Liver Transplant

**DOI:** 10.3389/fphar.2021.634084

**Published:** 2021-04-09

**Authors:** Roberto Minici, Michele Ammendola, Francesco Manti, Maria Anna Siciliano, Enrica Giglio, Marco Minici, Marica Melina, Giuseppe Currò, Domenico Laganà

**Affiliations:** ^1^ Radiology Unit, Department of Experimental and Clinical Medicine, Magna Graecia University, Catanzaro, Italy; ^2^ Digestive Surgery Unit, Science of Health Department, Magna Graecia University, Catanzaro, Italy; ^3^ Medical Oncology Unit, Department of Experimental and Clinical Medicine, Magna Graecia University, Catanzaro, Italy; ^4^ Medical Oncology Unit, University Hospital-Marche Polytechnic University, Ancona, Italy; ^5^ National Research Council (Cnr), Institute for High Performance Computing and Networking (ICAR), Rende, Italy; ^6^ Department of Medical and Surgical Sciences, Sant’Orsola Malpighi Hospital, Alma Mater Studiorum University of Bologna, Bologna, Italy; ^7^ General Surgery Unit, Science of Health Department, Magna Graecia University, Catanzaro, Italy

**Keywords:** bridging, transcatheter arterial chemoembolizalion, hepatocellar carcinoma, degradable starch microspheres, transarterial, doxorubicin, stromal microenvironment, tumoral angiogenesis

## Abstract

In patients with early-stage hepatocellular carcinoma, awaiting liver transplantation, current guidelines by AASLD and ESMO recommend a bridging therapy with a loco-regional treatment to prevent progression outside transplantation criteria. The standard of care in delaying disease progression has been recognized to be the transarterial chemoembolization. Permanent occlusion of tumor feeding vessels has effects on tumour stromal microenvironment by inducing intra- and intercellular signaling processes counteracting hypoxia, such as the release of vascular endothelial growth factor, a promoter of neoangiogenesis, tumour proliferation and metastatic growth. Among chemoembolization interventions, TACE with degradable starch microspheres represents an alternative to conventional cTACE and DEB-TACE and it minimizes detrimental effects on tumour stromal microenvironment, guaranteeing a transient occlusion of tumour feeding arteries and avoiding VEGF overexpression.Between January 2015 and September 2020, 54 consecutive patients with early-stage hepatocellular carcinoma and Child-Pugh stage B, who had undergone DSM-TACE as a bridging therapy while awaiting liver transplantation, were eligible for the study. A total of 154 DSM-TACE was performed, with a mean number of 2.85 procedures per patient. 18 patients (33.3%) succeeded in achieving liver transplantation, with a mean waiting time-to-transplantation of 11.7 months. The cumulative rates of patients still active on the WL at 6 months were about 91 and 93% when considering overall drop-out and tumour-specific drop-out respectively. Overall survival was about 96% at 6 months and 92% at 12 months. 17 patients experienced adverse events after the chemoembolizations. For patients with HCC in the transplant waiting list and within the Child-Pugh B stage, life expectancy may be dominated by the liver dysfunction, rather than by the tumour progression itself. In this population subset, the choice of LRT is critical because LRT itself could become a dangerous tool that is likely to precipitate liver dysfunction to an extent that survival is shortened rather than prolonged. Hence, the current study demonstrates that DSM-TACE is not far from being an ideal LRT, because it has an excellent safety profile, maintaining an efficacy that guarantees a clear advantage on the dropout rate with respect to the non-operative strategy, thus justifying its use.

## Introduction

In patients with very early and early-stage hepatocellular carcinoma (HCC), awaiting liver transplantation (LT), the disease may progress beyond transplantation criteria while on the waiting list. A transplant offers the benefit of cancer removal as well as the exclusion of the cirrhotic environment, which could have led to the emergence of new malignant lesions (Majno et al., 2011). Patients who develop tumour progression beyond the Milan criteria while awaiting liver transplantation become ineligible for an HCC MELD (Model for End-Stage Liver Disease) upgrade, which equates to waitlist drop out and subsequent death due to progression of HCC (Kulik et al., 2018). MELD exception points were introduced to alleviate dropouts due to tumour progression (Wiesner et al., 2004). Furthermore, locoregional treatments (LRTs) may prevent progression outside transplantation criteria (Harnois et al., 1999; Fontana et al., 2002; Lee et al., 2017) and EASL Guideline for the management of hepatocellular carcinoma defines bridging therapy as the treatment of accepted transplant candidates within Milan criteria while on the waiting list (Galle et al., 2018). Confirm that all author affiliations are correctly listed. Note that affiliations are listed sequentially as per journal style and requests for non-sequential listing will not be applied. Note that affiliations should reflect those at the time during which the work was undertaken). Current guidelines recommend a bridging therapy with LRT for patients within Milan criteria who are expected to remain on the transplant waitlist for more than 6 months, according to American guideline by AASLD (Heimbach et al., 2018), or for more than 3 months, according to European guideline by ESMO (Vogel et al., 2018). However, due to unpredictable waiting times and risk of tumour progression, most patients receive some form of LRT while awaiting transplant (Kulik et al., 2018).

The standard of care in delaying disease progression has been recognized to be the transarterial chemoembolization (TACE) (Llovet et al., 2002; Lo et al., 2002; Cescon et al., 2013). Despite several studies have shown controversial results (Hayashi et al., 2004; Tan et al., 2018), others have demonstrated advantages with a drop-out rate of 3–9.3% (Millonig et al., 2007; Alba et al., 2008), lower than those recorded without bridging therapies (7–11% at 6 months and ∼38% at 12 months) (Llovet et al., 1999a; Yao et al., 2002). Besides, TACE goes beyond its role of bridging therapy, as scientific evidence shows that patients who received TACE before LT had lower recurrence rates and improved overall survival (OS) (Millonig et al., 2007; Alba et al., 2008; Oligane et al., 2017). More thoroughly, TACE is beneficial when a complete or partial response can be achieved (Pompili et al., 2013), suggesting that response to LRT is a surrogate marker of tumour aggressive biology that may be used as a predictive factor to select patients in transplant waiting list (Mazzaferro et al., 1996; Yao et al., 2005; Sandow et al., 2018; Mazzaferro et al., 2016). This is supported by intention-to-treat studies who enlighten that downstaged patients have similar survival to patients inside criteria from the beginning (Ravaioli et al., 2008; Yao et al., 2015). However, stimulation of the immune system response may also explain the improved prognosis (Ayaru et al., 2007; Zerbini et al., 2010; Mizukoshi et al., 2011).

LRTs are an effective tool to minimize waitlist drop out but the selection of appropriate candidates is a non-negligible need to diminish the risk of exacerbating underlying liver disease and hence the development of worsening liver function and complications. A careful evaluation of advantages and disadvantages related to LRTs as bridging therapies is further necessary for patients with Child-Pugh stage B.

Among transarterial chemoembolization techniques, TACE with degradable starch microspheres (DSM) represents an alternative to conventional TACE (cTACE) with Lipiodol and chemotherapeutic agent or TACE with drug-eluting beads (DEB-TACE) (Gross and Albrecht, 2020). Carrying out a selective or super-selective catheterization with a complete embolization of Tumor Feeding Vessels (TFV) correlates with the efficacy of these treatments (Mauri et al., 2016). Permanent occlusion of TFV or an incomplete embolization has effects on tumour stromal microenvironment and induces intra- and intercellular signaling processes counteracting and reversing hypoxia (Carmeliet, 2005; Orlacchio et al., 2020), such as the activation of HIFs (hypoxia-inducible factors) and the subsequent release of vascular endothelial growth factor (VEGF), a promoter of neoangiogenesis, tumour proliferation and metastatic growth (Lencioni et al., 2013). To avoid VEGF overexpression and minimize detrimental effects on liver function, both induced by post-embolization ischemia, the idea of the transient occlusion of tumour feeding arteries (a half-life *in vitro* of 35–50 min) using DSM was born (Pieper et al., 2015; Schicho et al., 2016).

Despite the aforementioned rationale, data on the safety of DSM-TACE, in terms of tolerability and toxicity, are scarce but encouraging, showing a favourable trend in comparison with cTACE and DEB-TACE (Kirchhoff et al., 2006; Lammer et al., 2010; Niessen et al., 2014; Brown et al., 2016; Iezzi et al., 2016; Lencioni et al., 2016; Schicho et al., 2017; Gruber-Rouh et al., 2018; Orlacchio et al., 2018; Gross and Albrecht, 2020).

Hence, DSM-TACE is a powerful tool among transarterial chemoembolization techniques and it acts on the tumour-stromal microenvironment in combination with classic chemotherapeutic agents. Despite the absence of prospective comparative multicenter study, it shows a good safety profile in comparison with cTACE and DEB-TACE, that makes it an interestingly resource among LRT usable as bridge therapies in patients with HCC in transplant list, particularly in the population subset within Child-Pugh stage B, in which a huge focus to not worsening liver function should be paid and balance the evaluation of other parameters, such as the efficacy.

This study aims to define the safety and efficacy of DSM-TACE as a bridging therapy in patients with HCC and Child-Pugh stage B eligible for a liver transplant, attempting to cover a current lack of data regarding this technique applied to the aforementioned population subset.

## Materials and Methods

### Study Design

This study is a single-centre, retrospective analysis of prospectively collected data of consecutive patients with early stage hepatocellular carcinoma (HCC) and Child-Pugh stage B, who had undergone, from January 2015 to September 2020, DSM-TACE as a bridging therapy while awaiting liver transplantation (LT).

Inclusion criteria were: I) early stage (A) - according to the Barcelona-Clínic Liver Cancer (BCLC) staging system (Llovet et al., 1999b; Forner et al., 2010) - hepatocellular carcinoma, diagnosed with histological assessment or non-invasive imaging-based criteria used by European Association for the Study of the Liver (Galle et al., 2018); II) Child-Pugh stage B; III) age between 18 and 75 years; IV) no previous treatment for HCC; V) Eastern Cooperative Oncology Group performance status (Oken et al., 1982) grade 0; VI) registration on the transplant waiting list, fulfilling the Milan criteria (Mazzaferro et al., 1996); VII) evaluation by a multidisciplinary team of hepatologist, oncologist, liver surgeon and interventional radiologist. The exclusion criteria were: I) concomitant diseases not compatible with the transplantation; II) missed radiological evaluations at the follow-up; III) execution of liver resection or ablation during the follow-up; IV) serum creatinine levels >2.0 mg/dl; V) platelet count <50000/μL and/or international normalized ratio >1.5; VI) serum bilirubin level ≥3 mg/dl; VII) doxorubicin administration contraindications. The Institutional Review Board approval and informed written consent from each patient have been obtained.

### Intervention

At baseline condition, within 3 weeks before the first treatment, all patients underwent a clinical, biochemical and imaging examination. Imaging evaluation was performed with contrast-enhanced computed tomography (CT) and/or gadolinium-enhanced magnetic resonance imaging (MRI), using a multiphase liver imaging protocol.

DSM-TACE was performed within 2 weeks after the registration on the transplant waiting list, according to the evaluation made by a multidisciplinary team. DSM-TACE was performed in a dedicated angiography suite monitoring vital signs during anesthesia, by the same experienced interventional radiologists (30 and 2 years of experience, respectively). All patients were pre-medicated with a proton-pump inhibitor (Omeprazole 40 mg i.v.), a prokinetic drug (Metoclopramide 10 mg i.v.) and an analgesic drug (Ketorolac-Tromethamine 20 mg i.v.); if requested, conscious sedation was performed during the procedure. The treatment was performed through a femoral or radial approach, with a Seldinger needle, by using a 5-Fr arterial introducer sheath (Terumo, Tokyo, Japan). The selective celiac trunk catheterization and the cannulation of the common hepatic artery were performed with a 5-Fr diagnostic catheter (Cobra, Simmons; Terumo). The appropriate anatomy of the hepatic artery and any possible branches related to non-target structures and any possible arteriovenous fistulae were identified through a hepatic artery angiography. After diagnostic angiography, a selective lobar catheterization was performed with a coaxial technique, placing a 2.7-Fr microcatheter (Progreat; Terumo) in the right or left hepatic artery that was feeding the involved lobe. A selective lobar angiography was then performed to confirm the correct position of microcatheter, to identify non-hepatic arteries and limit any possible extrahepatic diffusion of the microspheres. In particular, the identification of the cystic artery was recommended to ensure that the catheter tip would bypass this anatomical point to avoid non-target embolization. When possible, a super-selective (segmental or sub-segmental) approach was obtained using the aforementioned microcatheter. However, when the selective catheterization of the feeding artery was not technically feasible, a lobar embolization, paying particular attention to prevent non-target embolization, was performed. DSMs were mixed with non-ionic iodinated contrast medium: 6 ml of nonionic iodinated contrast was used per 4 ml of DSMs before injection. Doxorubicin at a dose of 50 mg was diluted in 5 ml of normal saline. No dose adjustment was made for bilirubin concentration or body surface area. An appropriate suspension of DSMs, contrast medium and Doxorubicin was obtained before delivery. The mixture in the syringes was constantly shaken to avoid sedimentation and disaggregation of the microspheres, then slowly injected under fluoroscopic guidance at the proper site, until stasis was observed. Stasis was defined as the absence of antegrade flow within a vessel such that contrast filling the target vessel persisted, without washout, 5 cardiac beats after the injection of contrast (Brown et al., 2016). When stasis has been reached, a mixture of starch microspheres (4 ml) with contrast medium (6 ml) was slowly injected until a complete embolization was obtained.

All patients underwent physical examination, laboratory tests and imaging follow-up at 1 month after each treatment and every 3 months thereafter if no additional treatment was required. For each patient, the imaging modality (an abdominal contrast-enhanced CT or MRI examination) remained the same throughout the entire study period.

DSM-TACE treatments were repeated on-demand upon the demonstration of progressive or stable disease in patients who continued to meet the inclusion criteria until 1 of the following endpoints was reached: 1) CR or PR (OR); 2) technical impossibility to embolize the residual tumour, for example, in a tumour only supplied by extrahepatic collateral arteries; 3) development of contraindications to DSM-TACE; 4) total resection or ablation of the tumour by subsequent surgery or local ablation; 5) competing event for transplant list drop-out (liver transplantation or non-cancer-related death or refusing of LT); 6) PD after each of two consecutive DSM-TACE treatments; 7) worsening of at least 2 points of the Child-Pugh score. Causes of drop-out from transplant list were cancer-related death or cancer progression beyond the Milan Criteria.

### Outcomes

The primary efficacy endpoint was the time-to-event analysis (listing to drop-out), to examine the efficacy of DSM-TACE as bridging therapy in preventing drop-out from the waiting list (WL). Analyses were performed for both drop-outs as a result of all causes (HCC-specific and medical aetiologies) as well as tumour-specific drop-out (caused by cancer progression or cancer-related death), taking into account the competing events such as non-HCC-related mortality, liver transplantation and refusal of LT. Wait time was calculated from the date of listing until either transplant or delisting. For patients who developed HCC while on the list, wait time was adjusted to begin at the date of the diagnosis of HCC. Patients were censored at the end of the follow-up (September 30, 2020) or at the time of LT or at the time they refused transplant or at the time a noncancer-related death has occurred. A sub-group time to event analysis, to verify the relationships between lymphocyte-to-monocyte ratio (LMR)/neutrophil-to-Lymphocyte Ratio (NLR) and tumour-specific drop-out from WL, was performed. The secondary efficacy endpoints included the radiological response to treatment, the waiting time for LT, the overall survival, the progression-free survival and the proportion of patients transplanted. The overall survival (OS) was calculated as the time from the listing date until death or the last follow-up. The progression-free survival (PFS) was measured from the listing date to disease progression.

The primary safety endpoint was the incidence of serious adverse events (SAEs), in accordance with the classification set out in the next paragraph. The secondary safety endpoints were the incidence and severity of adverse events (AEs), including liver function parameters and laboratory abnormalities.

### Definitions

Technical success is defined as the ability to deliver the full planned dose of Doxorubicin and to obtain stop flow (Basile et al., 2012). Treatment response was assessed using mRECIST guidelines (Lencioni and Llovet, 2010). Complete response (CR) was defined as the disappearance of any intra-tumoral arterial enhancement in all target lesions. Partial response (PR) was defined as at least a 30% decrease in the sum of the diameters of viable (contrast-enhancing) target lesions. Progressive disease (PD) was defined as an increase of at least 20% in the sum of the diameters of the viable (enhancing) target lesions, and stable disease included all cases that did not qualify as either partial response or progressive disease. Patients developing new lesions, vascular invasion, and/or metastases were categorized as having PD. As previously reported (Lammer et al., 2010; Zhang et al., 2018), disease control (DC) was defined and calculated as CR + PR + SD. Responders referred to objective response (OR), namely the sum of patients who experienced CR or PR. Non-responders referred to the sum of patients who had stable disease (SD) and progressive disease (PD). The initial response was defined as the radiological response after the first DSM-TACE. The best response was defined as the best radiological response across repeated DSM-TACE sessions. Patients who achieved an objective response after the first treatment or after the following ones were considered as initial or best responders, respectively. Sustained response duration (SRD) was defined as the time between the date when CR, PR, or stable disease is achieved and the date progressive disease occurs.

All adverse events were graded using the National Cancer Institute Common Terminology Criteria for adverse events (CTCAE), version 4.0 (National Institute of Cancer, 2010), except for clinical complications associated with chemoembolization recorded using the CIRSE Classification System for Complications (Filippiadis et al., 2017). In reference to the CTCAE, toxicity was further graded using binary variables (mild: grades 1–2; serious: grades 3–4), adapted and modified from Kang et al., 2020.

### Statistical Analysis

Data were maintained in an Excel spreadsheet (Microsoft Inc., Redmond, Wash) and the statistical analyses were performed using SPSS software (SPSS, version 22 for Windows; SPSS Inc., Chicago IL, United States) and R/R Studio software. The analysis of efficacy was based on the Modified Intention-To-Treat (MITT) population, defined as all included patients who received at least one chemoembolization; this also defined the safety population. Kolmogorov-Smirnov test and Shapiro-Wilk test were used to verify the normality assumption of data. Categorical data are presented as frequency (percentage value). Continuous normally distributed data are presented as mean ± standard deviation. Continuous not normally distributed data are presented as median (interquartile range: 25th and 75th percentiles-IQR). The unpaired Student t-test was used to assess statistical differences for continuous normally distributed data, while categorical and continuous not normally distributed data were assessed using the Chi-squared test and the Mann-Whitney test, respectively. The incidence curves of tumour-specific drop-out were constructed and compared using the Gray method, taking into account the competing events (non-HCC-related mortality, liver transplantation and refusal of LT). Kaplan-Meier survival analysis was performed to assess time-dependent outcomes, and comparisons were made with the log-rank test. The independence between censored data and the tested events was assessed by clinical evaluation and telephone contacts in the cases of withdrawal. Hence, the assumption of independent censoring was met, avoiding bias regarding the observed time-dependent data. Among all survivors (with and without dropout from transplant list), follow-up was censored September 30, 2020. Univariate and multivariate analyses, using Cox proportional hazards and logistic regression models, were performed to identify individual predictors (patient/lesion characteristics) associated with drop-out while controlling for all other predictors in the model. A *p*-value of <0.05 was considered statistically significant for the aforementioned tests.

## Results

### Patient and Pathology Data

Between January 2015 and September 2020, 54 consecutive patients with early stage hepatocellular carcinoma (HCC) and Child-Pugh stage B, who had undergone DSM-TACE as a bridging therapy while awaiting liver transplantation (LT), were eligible for the study. All patients have received at least one chemoembolization treatment, meeting the criteria to be included in the Modified Intention-To-Treat (MITT) population. No patients were lost to follow-up. The mean age was 41.3 years and 77.8% of the patients were male. Among liver comorbidities, 11.1% of the patients had the hepatitis B virus, 40.7% the hepatitis C virus, 11.1% non-alcoholic fatty liver disease and 40.7% alcoholic liver disease. The median alpha-fetoprotein and Carbohydrate antigen 19–9 levels at the time of listing were 347 ng/ml and 9.7 U/ml, respectively. 38 patients (70.4%) were affected by cirrhosis; Child-Pugh score was B7 (88.9%) or B8 (11.1%). 59.3% of the patients had encephalopathy, while none had ascites. The median values of neutrophil-to-lymphocyte ratio (NLR) and lymphocyte-to-monocyte ratio (LMR) were 3.7 and 8.4, respectively. One-third of patients had one nodule, one-third of patients had two nodules and the other one third had three; the median (IQR) maximum tumour size was 2.5 cm (2.4–2.8 cm). A total of 18 patients (33.3%) had bilobar disease, while 36 patients (66.7%) have shown capsulated tumours.

Demographics and tumour data of the study population are reported in Table 1.

**TABLE 1:** Population data.

Variables		All patients (n = 54)
Age (years)–mean		41.3 (±16.6)
Sex (M/F)		42 (77.8%)/12 (22.2%)
Hepatitis B virus		6 (11.1%)
Hepatitis C virus		22 (40.7%)
Non-alcoholic fatty liver disease		6 (11.1%)
Alcoholic liver disease		22 (40.7%)
α-Fetoprotein (ng/ml)–median		347 (0–1370.8)
Carbohydrate antigen 19–9 (U/ml)–median		9.7 (0.7–24.7)
γ-Glutamyltransferase (U/L)–median		89 (2–176)
Alkaline phosphatase (U/L)–median		34 (9.8–58.2)
Aspartate transaminase (U/L)–median		30 (16–59.2)
Alanine transaminase (U/L)–median		43 (34.2–51.8)
Albumin (g/L)–median		27 (24–31)
Total bilirubin (mg/dl)–median		1.0 (0.8–1.4)
Prothrombin time (seconds prolonged)–median		8 (7–9)
Ascites, no/yes		54 (100%)/0 (0%)
Encephalopathy, no/yes		32 (59.3%)/22 (40.7%)
Child-Pugh score, B7/B8		48 (88.9%)/6 (11.1%)
Cirrhosis, no/yes		16 (29.6%)/38 (70.4%)
Platelet count (no. x10^3^/μl)–median		96 (62.2–129.8)
Creatinine (mg/dl)–median		1.2 (1.1–1.3)
Hemoglobin (g/dl)–median		13.5 (13.1–14.1)
White blood cell count (per μL)–median		4009 (4001–4230)
	Neutrophil count (per μL)	3009 (2989–3202)
	Lymphocyte count (per μL)	809 (698–898)
	Monocyte count (per μL)	108 (81–199)
	Neutrophil-to-lymphocyte ratio (NLR)	3.7 (3.3–7.3)
	Lymphocyte-to-monocyte ratio (LMR)	8.4 (3.5–9.9)
Number of Tumors, 1/2/3		18 (33.3%)/18 (33.3%)/18 (33.3%)
Maximum tumour size (cm)–median		2.5 (2.4–2.8)
Bilobar disease, no/yes		36 (66.7%)/18 (33.3%)
Capsule, absent/present		18 (33.3%)/36 (66.7%)

### Procedure Data

A total of 154 DSM-TACE was performed, with a mean number of 2.85 procedures per patient. The chemoembolization pattern was selective in 119 procedures (77.2%) and lobar in 35 procedures (22.8%); no procedure was performed with the catheter placed in the common hepatic artery.

Procedure data are reported in Table 2.

**TABLE 2:** Procedure and Outcomes data.

Variables		All patients (n = 54)
Total number of DSM-TACEs		154
Mean number of DSM-TACEs per patient		2.85
Mean follow-up (months)		23.7
Chemoembolization pattern		
	Selective/Superselective	119 (77.2%)
	Lobar	35 (22.8%)
	Global	0
Technical success, no/yes		0 (0%)/154 (100%)
Tumour response to first DSM-TACE (no.)		54
	CR	4 (7.4%)
	PR	12 (22.2%)
	SD	30 (55.6%)
	PD	8 (14.8%)
	Non-responders (SD + PD)	38 (70.4%)
	Responders or OR (CR + PR)	16 (29.6%)
	DC (CR + PR + SD)	46 (85.1%)
Tumour response to second DSM-TACE (no.)		42
	CR	2 (4.8%)
	PR	10 (23.8%)
	SD	16 (38.1%)
	PD	14 (33.3%)
	Non-responders (SD + PD)	30 (71.4%)
	Responders or OR (CR + PR)	12 (28.6%)
	DC (CR + PR + SD)	28 (66.7%)
Tumour response to third DSM-TACE (no.)		38
	CR	0 (0%)
	PR	14 (36.8%)
	SD	10 (26.3%)
	PD	14 (36.9%)
	Non-responders (SD + PD)	24 (63.2%)
	Responders or OR (CR + PR)	14 (36.8%)
	DC (CR + PR + SD)	24 (63.2%)
Tumour response to fourth DSM-TACE (no.)		20
	CR	0 (0%)
	PR	0 (0%)
	SD	6 (30%)
	PD	14 (70%)
	Non-responders (SD + PD)	20 (100%)
	Responders or OR (CR + PR)	0 (0%)
	DC (CR + PR + SD)	6 (30%)
Best Response (no.)		54
	CR	6 (11.1%)
	PR	32 (59.3%)
	SD	12 (22.2%)
	PD	4 (7.4%)
	Non-responders (SD + PD)	16 (29.6%)
	Responders or OR (CR + PR)	38 (70.4%)
	DC (CR + PR + SD)	50 (92.6%)
Sustained Response duration (SRD), <6 months/≥6 months		28 (51.8%)/26 (48.2%)
Time-to-dropout from transplant list (months) *- mean*		14.7 (±7.6)
Event, censoring/death		26 (48.1%)/28 (51.9%)
Event, censoring/Hcc-related dropout		38 (70.4%)/16 (29.6%)
Event, censoring/Overall dropout		29 (53.7%)/25 (46.3%)
Liver Transplantation, no/yes		36 (66.7%)/18 (33.3%)
Waiting time-to-transplantation (months) *- mean*		11.7 (±4.6)
Post-procedural clinical complications (CIRSE class.), absent/present		38 (70.4%)/16 (29.6%)
	Grade 1	14 (25.9%)
	Grade 2	0 (0%)
	Grade 3	2 (3.7%)
Adverse Events (CTCAE), absent/present		37 (68.5%)/17 (31.5%)
	Grade 1	9 (16.7%)
	Grade 2	6 (11.1%)
	Grade 3	2 (3.7%)
	Grade 4	0 (0%)
	Serious Adverse Events	2 (3.7%)

### Efficacy Outcomes

Technical success was achieved in 154 procedures (100%). The average follow-up was 23.7 months. After the first DSM-TACE, CR was achieved in 4 of 54 patients (7.4%), PR in 12 (22.2%), SD in 30 (55.6%) and PD in 8 (14.8%), with 38 (70.4%) non-responders, 16 (29.6%) responders (OR) and disease control (DC) achieved after 46 (85.1%) procedures. A second DSM-TACE was performed in 42 cases, after which CR was achieved in 2 patients (4.8%), PR in 10 (23.8%), SD in 16 (38.1%) and PD in 14 (33.3%). A third DSM-TACE was performed in 38 cases, after which no CR was achieved, PR was achieved in 14 patients (36.8%), SD in 10 (26.3%) and PD in 14 (36.9%). A fourth DSM-TACE was performed in 20 cases, after which no CR and PR were achieved, SD was achieved in 6 patients (30%) and PD in 14 (70%). Considering the best response across repeated DSM-TACE sessions for each patient, CR was achieved in 6 of 54 patients (11.1%), PR in 32 (59.3%), SD in 12 (22.2%) and PD in 4 (7.4%), with 16 (29.6%) non-responders, 38 (70.4%) responders (OR) and disease control (DC) achieved in 50 patients (92.6%). 26 (48.2%) patients achieved a Sustained Response Duration (SRD) of 6 months or more; the rest of the patients (51.8%) achieved an SRD of fewer than 6 months. Overall drop-out from WL was observed in 25 (46.3%) cases, while tumour-specific drop-out was observed only in 16 (29.6%) cases. The mean time-to-dropout from transplant waiting list was 14.7 (±7.6) months, considering Hcc-related drop-out. 18 patients (33.3%) succeeded in achieving liver transplantation, with a mean waiting time-to-transplantation of 11.7 (±4.6) months. When considering overall drop-out, the cumulative rates of patients still active on the WL were about 91% (±0.04) at 6 months, 72% (±0.07) at 12 months and 33% (±0.09) at 24 months. When considering tumour-specific drop-out, the cumulative rates of patients still active on the WL were about 93% (±0.04) at 6 months, 78% (±0.06) at 12 months and 52% (±0.10) at 24 months. For patients with lymphocyte-to-monocyte ratio (LMR) ≥ 4 and neutrophil-to-lymphocyte ratio (NLR) < 7.2, the median (range) time-to-dropout from WL was 23 (21-NA) months, which was better than that of patients with lymphocyte-to-monocyte ratio (LMR) < 4 and neutrophil-to-lymphocyte ratio (NLR) ≥ 7.2 (median [range] time-to-dropout from WL, 12 [9–19] months) (*p* = 0.00013, calculated by mean of Log-Rank test). The death occurred in 28 cases (51.9%) along the follow-up period. OS was about 96% (±0.03) at 6 months, 92% (±0.04) at 12 months and 48% (±0.07) at 24 months. For patients with SRD of more than 6 months, the median (range) OS was not applicable but still greater than 36 months (21-NA) months, which was better than that of patients with SRD of less than 6 months (median [range] OS, 21 [17-NA] months), although not statistically significant (*p* = 0.086, calculated by mean of Log-Rank test). Progression-free survival (PFS) was about 70% (±0.06) at 6 months, 51% (±0.07) at 12 months and 14% (±0.06) at 24 months with only 4 residual patients at risk.

**FIGURE 1:**
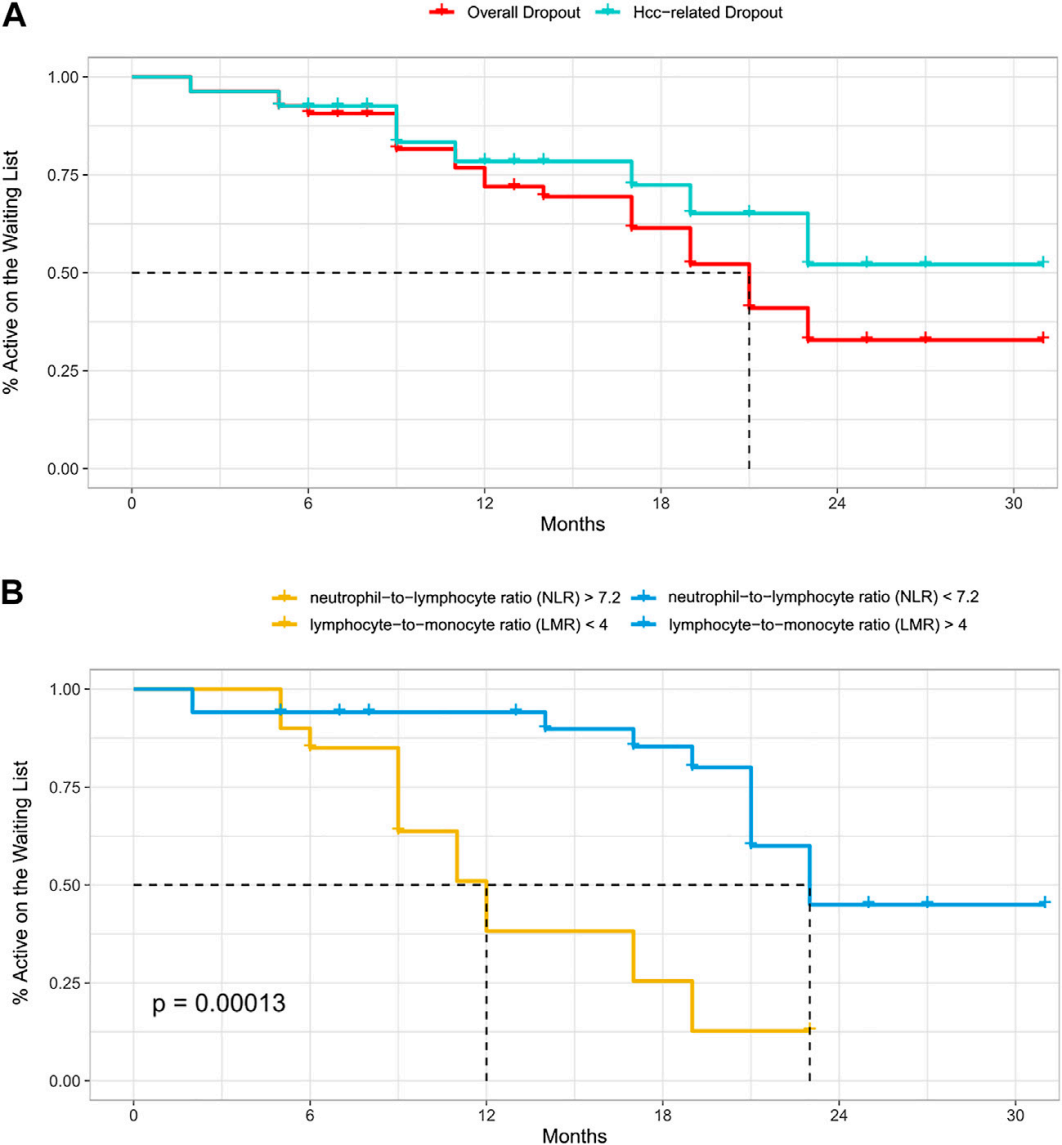
**(A–B)** Time-to-event analysis (listing to drop-out), according to the causes of drop-out (tumour-specific or all-causes) **(A)** and to the neutrophil-to-lymphocyte/lymphocyte-to-monocyte ratios in case of tumour-specific drop-out **(B)**.

**FIGURE 2:**
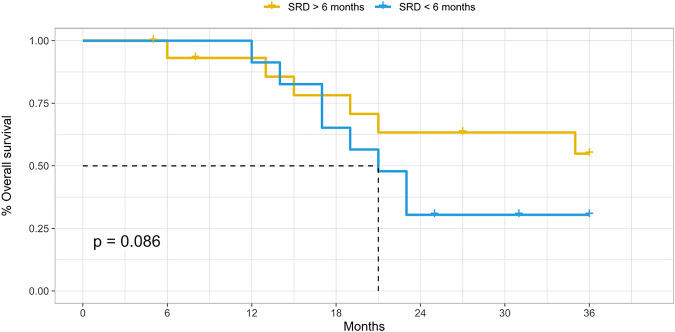
Time-to-event analysis (listing to death), according to the Sustained Response Duration (SRD).

The efficacy outcomes are shown in Tables 2, 3, 4; Figures 1, 2.

**TABLE 3:** Time-to-event outcomes (listing to drop-out) as indicated in the related survival plot (Figure 1).

	Cumulative rates of patients active on the WL	At 6 months rate (±SE)-numbers at risk	At 12 months rate (±SE)-numbers at risk	At 24 months rate (±SE)-numbers at risk
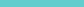	Hcc-related dropout	93% (±0.04)–48	78% (±0.06)–32	52% (±0.10)–6
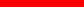	Overall dropout	91% (±0.04)–48	72% (±0.07)–32	33% (±0.09)–6
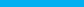	LMR >4/NLR <7.2	94% (±0.04)–30	94% (±0.04)–24	45% (±0.13)–6
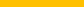	LMR <4/NLR >7.2	85% (±0.08)–18	38% (±0.12)–8	NA

**TABLE 4:** Time-to-event outcomes (listing to death/disease progression) as partly indicated in the related survival plot (Figure 2).

	Cumulative rates	At 6 months rate (±SE) -numbers at risk	At 12 months rate (±SE)-numbers at risk	At 24 months rate (±SE)-numbers at risk
	Overall Survival (OS)	96% (±0.03)–52	92% (±0.04)–48	48% (±0.07)–24
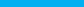	OS according to SRD < 6 m	100% (±0.00)–23	91% (±0.06)–23	30% (±0.10)–7
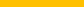	OS according to SRD > 6 m	93% (CT±0.05)–29	93% (±0.05)–25	63% (±0.09)–17
	Progression-free Survival (PFS)	70% (±0.06)–42	51% (±0.07)–24	14% (±0.06)–4

Details of the predictors of drop-out are listed in Table 5. Based upon the intention to treat on both univariate and multivariate analysis, aspartate transaminase more than 40 U/L (HR, 1.3; 95% CI, 1.2–1.6; *p* 0.04), number of tumors equal or less than two (HR, 0.6; 95% CI, 0.4–1.1; *p* 0.01), presence of tumor capsule (HR, 0.6; 95% CI, 0.3–1.0; *p* 0.01), objective response as the best response across repeated DSM-TACE sessions for each patient (HR, 0.6; 95% CI, 0.4–1.2; *p* 0.01), objective response as the initial response (HR, 0.5; 95% CI, 0.4–0.9; *p* 0.01), SRD of 6 months or more (HR, 0.3; 95% CI, 0.2–0.9; *p* 0.01) and lymphocyte-to-monocyte ratio (LMR) ≥ 4/neutrophil-to-lymphocyte ratio (NLR) < 7.2 (HR, 0.4; 95% CI, 0.3–0.7; *p* 0.01) were found to be the independent prognostic factors for drop-out from waiting list.

**TABLE 5:** Factors predicting drop-out from the waiting list.

Variable	Univariate HR (95%CI)–*p* value	Multivariate HR (95%CI)–*p* value
Age	1.2 (0.9–1.3)–0.59	NA
Age ≥60 years	1.3 (1.0–1.5)–0.53	NA
Sex (Male)	0.9 (0.7–1.3)–0.81	NA
Hepatitis B virus	1.1 (0.9–1.5)–0.79	NA
Hepatitis C virus	1.3 (1.0–1.5)–0.53	NA
Non-alcoholic fatty liver disease	1.2 (0.8–1.3)–0.61	NA
Alcoholic liver disease	1.2 (1.1–2.1)–0.39	NA
α-Fetoprotein (ng/ml) > 300	0.9 (0.7–1.3)–0.80	NA
Aspartate transaminase (>40 U/L vs ≤ 40)	1.4 (1.3–1.6)–0.04	1.3 (1.2–1.6)–0.04
Alanine transaminase (>40 U/L vs ≤ 40)	1.1 (1.0–1.3)–0.45	NA
Cirrhosis (yes vs no)	0.8 (0.7–1.1)– 0.08	NA
Tumour no. (≤2 vs 3)	0.6 (0.4–1.2)–0.01	0.6 (0.4–1.1)–0.01
Capsule (present vs absent)	0.6 (0.5–1.1)–0.01	0.6 (0.3–1.0)–0.01
Objective response as the best response	0.6 (0.4–1.2)–0.01	0.6 (0.4–1.2)–0.01
Objective response as the initial response	0.5 (0.3–0.9)–0.01	0.5 (0.4–0.9)–0.01
SRD (≥6 months vs < 6 months)	0.5 (0.3–1.0) –0.01	0.3 (0.2–0.9)–0.01
LMR/NLR (≥4/< 7.2 vs. < 4/≥ 7.2)	0.4 (0.3–0.8)–0.01	0.4 (0.3–0.7)–0.01

### Safety Outcomes

According to the CIRSE Classification System for Complications, 16 patients (29.6%) experienced postprocedural clinical complications associated with chemoembolization. Apart from two treatment-related grade 3 events (non-surgical cholecystitis), only grade 1 events occurred (14 cases, 25.9%). These were pain responsive to analgesics (8 DSM-TACEs, 14.9%), post-embolization syndrome (4 DSM-TACEs, 7.4%), transient nausea (1 DSM-TACEs, 1.8%) and vomiting (1 DSM-TACEs, 1.8%). The aforementioned adverse events were transient and easily solved with standard analgesic or antiemetic medication during interventions.

According to the CTCAE classification, 17 patients (31.5%) experienced adverse events after the chemoembolizations. Grade 1 events were observed in 9 of 54 patients (16.7%), grade 2 events in 6 (11.1%) and grade 3 in 2 (3.7%); no grade 4 adverse events were observed. Hence, only two (3.7%) serious adverse events (SAE) occurred, namely, grade 3 or 4 adverse events according to the Common Terminology Criteria for adverse events (CTCAE).

Details are given in Table 2.

## Discussion

Current guidelines recommend a bridging therapy with LRT for patients with HCC within Milan criteria who are expected to remain on the transplant waitlist for more than 6 months, according to American guideline by AASLD (Heimbach et al., 2018), or for more than 3 months, according to European guideline by ESMO (Vogel et al., 2018). The standard of care in delaying disease progression has been recognized to be the transarterial chemoembolization (TACE) (Llovet et al., 2002; Lo et al., 2002; Cescon et al., 2013). Among chemoembolization interventions, despite the absence of prospective comparative multicenter study, DSM-TACE shows an excellent safety profile in comparison with cTACE and DEB-TACE and a non-inferior efficacy (Kirchhoff et al., 2006; Lammer et al., 2010; Niessen et al., 2014; Brown et al., 2016; Iezzi et al., 2016; Lencioni et al., 2016; Schicho et al., 2017; Gruber-Rouh et al., 2018; Orlacchio et al., 2018; Gross and Albrecht, 2020). For patients with HCC in the transplant waiting list and within Child-Pugh B stage, life expectancy may be dominated by the liver dysfunction, rather than by the tumour progression itself (Bolondi et al., 2012). In this population subset, the choice of LRT is critical because LRT itself could become a dangerous tool that is likely to precipitate liver dysfunction to an extent that survival is shortened rather than prolonged. Hence, the ideal LRT used as bridging therapy in patients with hepatocellular carcinoma within Child-Pugh stage B awaiting liver transplantation should have an excellent safety profile, maintaining an efficacy that guarantees a clear advantage on the dropout rate, thus justifying its use. Based on the aforementioned rationale, DSM-TACE could prove to be an interesting tool. The primary efficacy endpoint was the time-to-event analysis (listing to drop-out) and the cumulative rates of patients still active on the WL at 6 months were about 91% (±0.04) and 93% (±0.04), when considering overall drop-out and tumour-specific drop-out respectively. These results seem to be at least comparable with those reported by Kulik in his meta-analysis on LRTs used as bridging therapies (Kulik et al., 2018), in which dropout rates due to all causes and due to progression were 0.19 (95% CI 0.15–0.24) and 0.11 (95% CI 0.07–0.17) respectively. For patients with lymphocyte-to-monocyte ratio (LMR) ≥ 4 and neutrophil-to-lymphocyte ratio (NLR) < 7.2, the median time-to-dropout from WL was significantly better than that of patients with lymphocyte-to-monocyte ratio (LMR) < 4 and neutrophil-to-lymphocyte ratio (NLR) ≥ 7.2. The prognostic role of neutrophil-to-lymphocyte ratio (NLR) in certain cancer populations have already been investigated, also in the HCC (Dan et al., 2013), but, at the best of our knowledge, no data on the lymphocyte-to-monocyte ratio (LMR) in HCC patients have been published so far. Therefore, NLR and LMR together could act as a marker that reflects the balance between host inflammatory response, which gives a major contribution to tumour-related angiogenesis, and immune response, which has a pivotal role in cytotoxic cancer cells death. Patients with elevated preoperative NLR and low preoperative LMR have poorer dropout rates, therefore these ratios could be used as surrogate markers of tumour aggressiveness, suggesting a more aggressive bridging strategy while on the waiting list for liver transplantation. The mean waiting time-to-transplantation (11.7 months) was similar to that reported for radiofrequency ablation (RFA) used as bridging therapy (9.5 months) (DuBay et al., 2010), with one-third of patients enrolled (18 patients, 33%) successfully transplanted at the end of the follow-up (mean follow-up time of 23.7 months). Response to loco-regional therapy may be a surrogate of tumour aggressiveness and has been reported to correlate with post LT outcomes. Furthermore, a period of at least 6 months of sustained response duration (SRD) after a successful downstaging was found to be an independent prognostic factor for OS, even after liver transplantation (Zhang et al., 2018). In our study, patients with SRD of more than 6 months have shown a favourable trend in OS, although not statistically significant (*p* = 0.086, calculated by means of Log-Rank test), compared to patients with SRD of less than 6 months. Besides, multivariate analysis showed that the dropout risk of patients with SRD of 6 months or more was reduced by 70%, more than that of initial responders (50%) and best responders (40%). Interestingly, these data demonstrate that among patients with an objective response, such as CR or PR, some factors other than radiological response, such as tumour biology and tumour microenvironment, may alter the efficacy of TACE. This implies that the maintenance of response, rather than achieving the radiological Objective Response (OR) itself, maybe more clinically important for long-term outcomes because more related to histological tumour necrosis. Hence, SRD reflects the result of the interactions between tumour cells, liver disease biology, and tumour microenvironment, and can be used in clinical practice as an excellent and reliable predictor of outcomes, similar to that shown by Zhang (Zhang et al., 2018). To date, no studies have previously explored the role of sustained response duration (SRD) in predicting clinical prognosis of early stage HCC after TACE in a bridging therapy setting. Moreover, the overall survival was equivalent, if not with a favourable trend (92% at 12 months), to that published in the literature for RFA performed as bridging therapy (Dubay et al., 2011). Therefore, DSM-TACE, when used as bridging therapy in preventing drop-out from the waiting list (WL), has shown comparable efficacy outcomes to those recorded with other LRTs. Safety of DSM-TACE in the present study is comparable to previous data on cTACE, DEB-TACE, DSM-TACE and RFA (DuBay et al., 2011; Gross and Albrecht, 2020; Iezzi et al., 2016; Lammer et al., 2010; Brown et al., 2016; Lencioni et al., 2016). The incidence of adverse events (31.5%) and serious adverse events (3.7%) after the chemoembolization procedures was very low, resulting in an excellent safety profile despite the high-risk population subset of Child-Pugh B patients.

Limitations of the study are the lack of a control group, the single-centre setting, the retrospectivity of the analysis and the scarcity of data in the literature, necessary to evaluate the congruence and the consistency of the data presented. Besides, patients with SRD of more than 6 months have shown a favourable trend in OS, although not statistically significant (*p* = 0.086, calculated by means of Log-Rank test), compared to patients with SRD of less than 6 months; the absence of statistical significance may be due to a lack of statistical power, therefore a type II statistical error cannot be excluded. Good response to locoregional treatments in patients awaiting LT is a surrogate marker of favourable tumour biology and correlates with long-term OS after LT (Millonig et al., 2007). Hence, making a per-protocol analysis could lead to an unintentional selection bias, selecting a priori a population subset with less aggressive disease biology and favourable drop-out percentage and long-term post-LT OS. The modified-intention-to-treat analysis performed in this study should have minimized the aforementioned risk.

## Conclusion

At the best of our knowledge, no observational studies have so far verified the efficacy and safety profile of DSM-TACE as bridging therapy in the population subset of patients with HCC and Child-Pugh stage B, eligible for liver transplantation. Hence, the results of the current study demonstrate that DSM-TACE could be an ideal LRT, because it has an excellent safety profile, maintaining an efficacy that guarantees a clear advantage on the dropout rate with respect to the non-operative strategy, thus justifying its use.

Furthermore, sustained response duration (SRD) has a pivotal role in predicting clinical prognosis of early stage HCC after TACE in a bridging therapy setting and neutrophil-to-lymphocyte/lymphocyte-to-monocyte ratios (NLR and LMR) could be used as surrogate markers of tumour aggressiveness; how they will practically guide the bridging strategy needs to be furtherly investigated.

## Data Availability

The raw data supporting the conclusions of this article will be made available by the authors, without undue reservation.

## Abbreviations

AASLD, American association for the study of liver diseases; AEs, adverse events; BCLC, Barcelona-clínic liver cancer; CT, computed tomography; cTACE, conventional transarterial chemoembolization; DC, disease control; DEB, drug-eluting beads; DSM, degradable starch microspheres; ESMO, European society for medical oncology; HCC, hepatocellular carcinoma; HIFs, hypoxia-inducible factors; LMR, lymphocyte-to-monocyte ratio; LRTs, locoregional treatments; LT, liver transplantation; MELD, model for end-stage liver disease; MITT, modified intention-to-treat; NLR, neutrophil-to-lymphocyte ratio; OR, objective response; OS, overall survival; PFS, progression-free survival; RFA, radiofrequency ablation; SAEs, serious adverse events; SRD, sustained response duration; TACE, transarterial chemoembolization; TFV, tumor feeding vessel; VEGF, vascular endothelial growth factor; WL, waiting list
